# Tracing the acid-base catalytic properties of MO*N*_2_O mixed oxides (M = Be, Mg, Ca; *N* = Li, Na, K) by theoretical calculations

**DOI:** 10.1007/s00894-021-04829-7

**Published:** 2021-06-26

**Authors:** Dawid Faron, Piotr Skurski, Iwona Anusiewicz

**Affiliations:** Laboratory of Quantum Chemistry, Faculty of Chemistry, University of Gdańsk, Wita Stwosza 63, 80-308 Gdańsk, Poland

**Keywords:** Mixed oxides, Gas-phase basicity, Gas-phase electrophilicity, Proton affinity, Hydride affinity

## Abstract

The stability and acid-base properties of **M**O***N***_2_O mixed oxides (where **M** = Be, Mg, Ca; ***N*** = Li, Na, K) are studied by using ab initio methods. It is demonstrated that (i) the basicity of such designed systems evaluated by estimation of electronic proton affinity and gas-phase basicity (defined as the electronic and Gibbs free energies of deprotonation processes for [**M**O***N***_2_O]H^+^) were found significant (in the ranges of 272–333 and 260–322 kcal/mol, respectively); (ii) in each series of **M**OLi_2_O/**M**ONa_2_O/**M**OK_2_O, the basicity increases with an increase of the atomic number of alkali metal involved; (ii) the Lewis acidity of the corresponding [**M**O***N***_2_O]H^+^ determined with respect to hydride anion (assessed as the electronic and Gibbs free energies of H^−^ detachment processes for [**M**O***N***_2_O]H_2_) decreases as the basicity of the corresponding oxide increases. The thermodynamic stability of all [**M**O***N***_2_O]H_2_ systems is confirmed by estimating the Gibbs free energies for the fragmentation processes yielding either H_2_ or H_2_O.

## Introduction

The alkaline earth metal oxides are classical base catalysts where oxide ions behave as bases whereas the metal cations serve as Lewis acids. They catalyse a variety of organic reactions, e.g. isomerization of olefins[1], aldol condensation[2–6], transesterification reactions [7–11], the Knoevenagel condensation[12, 13], the Michael addition[14–16], dehydrogenation reactions[17–19] and many other processes which require the cleavage of the C–H bond and the formation of carbanion intermediates[20, 21]. For modern industrial applications, a good catalyst is the catalyst which is relatively inexpensive, easily accessible and, most importantly, environmental friendly. In many existing processes which use homogeneous catalysts, the removal of catalysts after the reaction is usually a difficult task and a large amount of liquid waste is produced. Among the different fields of catalysis, the heterogeneous catalysis utilizing metal oxides is very prominent in the context of improving industrial processes that fulfil the needs of sustainable technologies (regulated by environmental issues)[22]. In the case of solid catalysts, many important parameters or features act on catalytic properties, such as (i) atomic composition (i.e. the presence of transition metals or main group elements only), (ii) the structure of crystalline phase, (iii) the surface morphology (i.e. isotropic, anisotropic or amorphous) and (iv) structural defects[23]. As stated above, the solid alkaline earth metal oxides (**M**O) are bifunctional which means they possess two active sites (i.e. the M^2+^ cation and the O^2−^ anion). Therefore, the catalytic activity may be also attributed to acid-base strength of **M**O. The acid-base strength is especially important in the organic reactions mentioned above. Namely, the stronger the basic site of the metal oxide catalyst, the faster the cleavage of the C–H bond, while the low Lewis acidity strength reduces the activation barrier related to the formation of carbanion-catalyst complex which in turn increases its reactivity. In this contribution, we present our theoretical study of the structure and acid-base properties of **M**O***N***_2_O mixed oxides (where **M** = Be, Mg, Ca; ***N*** = Li, Na, K). Our goal was to investigate whether the potential catalytic properties (in terms of theoretically predicted acid-base properties) of alkaline earth metal oxides can be enhanced by combining with alkali metal oxides. The introduction of different metals (i.e. alkali or alkali earth metals) into the structure of solid transition metal catalyst (including transition metal oxides) is one of the ways to enhance either the selectivity or activity of the catalyst[24]. The promotion effect of dopants depends on the metal used and usually improves the active sites of a catalyst by changing its physicochemical properties. For instance, during the N_2_O decomposition reaction, strong promotion effects of alkali metals on cobalt-cerium composite oxide were observed [25]. The high catalytic activity was attributed to the redox ability of active Co^2+^ site induced by alkali metal. This is consistent with our results reported for mixed nonstoichiometric **M**O***N*** oxides (where **M** = Be, Mg, Ca; ***N*** = Li, Na, K) [26]. We found that the introduction of alkali metal to alkaline earth metal oxide substantially affects the electron density distribution in the **M**O system (by reducing the partial charge on alkali earth metal atom) and rises the reductive ability (by ca. 2–3 eV with respect to the unmodified oxide).

In considering the potential applicability of base catalysts (pure or modified), it is convenient to be able to characterize their activity in terms of the number of sites and the strength thereof. It is being experimentally accomplished by the use of numerous technics, such as the usage of acid-base indicators, X-ray diffraction, photoelectron spectroscopy or thermal analysis [23]. On the other hand, the intrinsic basicity of any molecule can be estimated theoretically by performing ab initio calculations. The calculated values of proton affinity (PA) and the negative of the Gibbs free energy of protonation reaction (known as gas-phase basicity, GPB) have been determined for a large number of species and are available through the NIST chemistry webbook[27]. It is worth noting that among the neutral systems one of the strongest basis proposed thus far is the bidentate proton chelator “proton sponge” (1,8-bis(dimethylamine)naphthalene) whose PA and GPB were estimated to be equal to ca. 245 and 239 kcal/mol, respectively[28]. In fact, many chemists adopt those values as the threshold values while classifying compounds as “superbases” (defined as compounds whose proton affinity and gas-phase basicity are both larger than that of the “proton sponge”). As far as the second (acidic) site is concerned, its strength might be estimated by its vulnerability to accept the electron pair (as this site is to promote the carbanion). Therefore, in our contribution, we decided to relate such property to hydride anion bounding strength. Namely, we calculated the hydride affinity (HA) and the Gibbs free energy of hydride anion detachment process (so-called gas-phase electrophilicity, GPE) by analogy with the PA and GPB values. To the best of our knowledge, this is the first report containing a systematic study of the acid-base properties of **M**O***N***_2_O mixed oxides and their physicochemical properties with respect to chemical composition.

## Methods

The equilibrium structures of the **M**O, (**M**O)_2_, **M**O***N***_2_O, [**M**O]H^+^, [(**M**O)_2_]H^+^ and [**M**O***N***_2_O]H^+^, [**M**O]H_2_, [(**M**O)_2_]H_2_ and [**M**O***N***_2_O]H_2_ (where **M** = Be, Mg, Ca; ***N*** = Li, Na, K) molecules and the corresponding harmonic vibrational frequencies were calculated using the second-order Møller-Plesset perturbational method (MP2)[29–31] with the aug-cc-pVTZ basis set[32, 33]. The electronic energies of the systems studied were then refined by employing the coupled-cluster method with single, double and noniterative triple excitations (CCSD(T))[34–37] and the same basis set. During the geometry optimizations followed by harmonic vibrational frequency calculations carried out by employing the MP2 method and while refining the electronic energies using the CCSD(T) method, all orbitals in the core and valence shells have been correlated.

The electronic proton affinity (PA, defined as the negative of the electronic energy change in the reaction B+H^+^ → BH^+^) of **M**O, (**M**O)_2_ and **M**O***N***_2_O and electronic hydride affinity (HA, defined as the negative of the electronic energy change in the reaction BH^+^+H^–^→BH_2_) of the **M**OH^+^, [(**M**O)_2_]H^+^, [**M**O***N***_2_O]H^+^ were estimated at the CCSD(T)/aug-cc-pVTZ theory level. The corresponding gas-phase basicity (GPB, defined as the negative of the Gibbs free energy of protonation reaction) of **M**O, (**M**O)_2_ and **M**O***N***_2_O and gas-phase electrophilicity (GPE, defined as the negative of the Gibbs free energy of hydride anion attachment) of the [**M**O]H^+^, [(**M**O)_2_]H^+^, [**M**O***N***_2_O]H^+^ were evaluated using the CCSD(T)/aug-cc-pVTZ electronic energies and the zero-point energy corrections, thermal corrections (at *T* = 298.15K) and entropy contributions estimated with the MP2 method and the aug-cc-pVTZ basis set (in each case, the Gibbs free energy of a proton was also accounted for).

Thermodynamic stability related to the most likely fragmentations paths for all studied species was established accordingly, by using the CCSD(T) electronic energies and the MP2 zero-point energies, thermal corrections and entropy contributions (at *T* = 298.15K).

All calculations were performed with the GAUSSIAN16 program (*Rev. C.01*)[38], while the plots showing the molecular structures were generated with the CHEMCRAFT program[39]

## Results

### The [**M**O]H^+^, [(**M**O)_2_]H^+^, [**M**O]H_2_ and [(**M**O)_2_]H_2_ systems (**M** = Be, Mg, Ca)

**Fig. 1:**
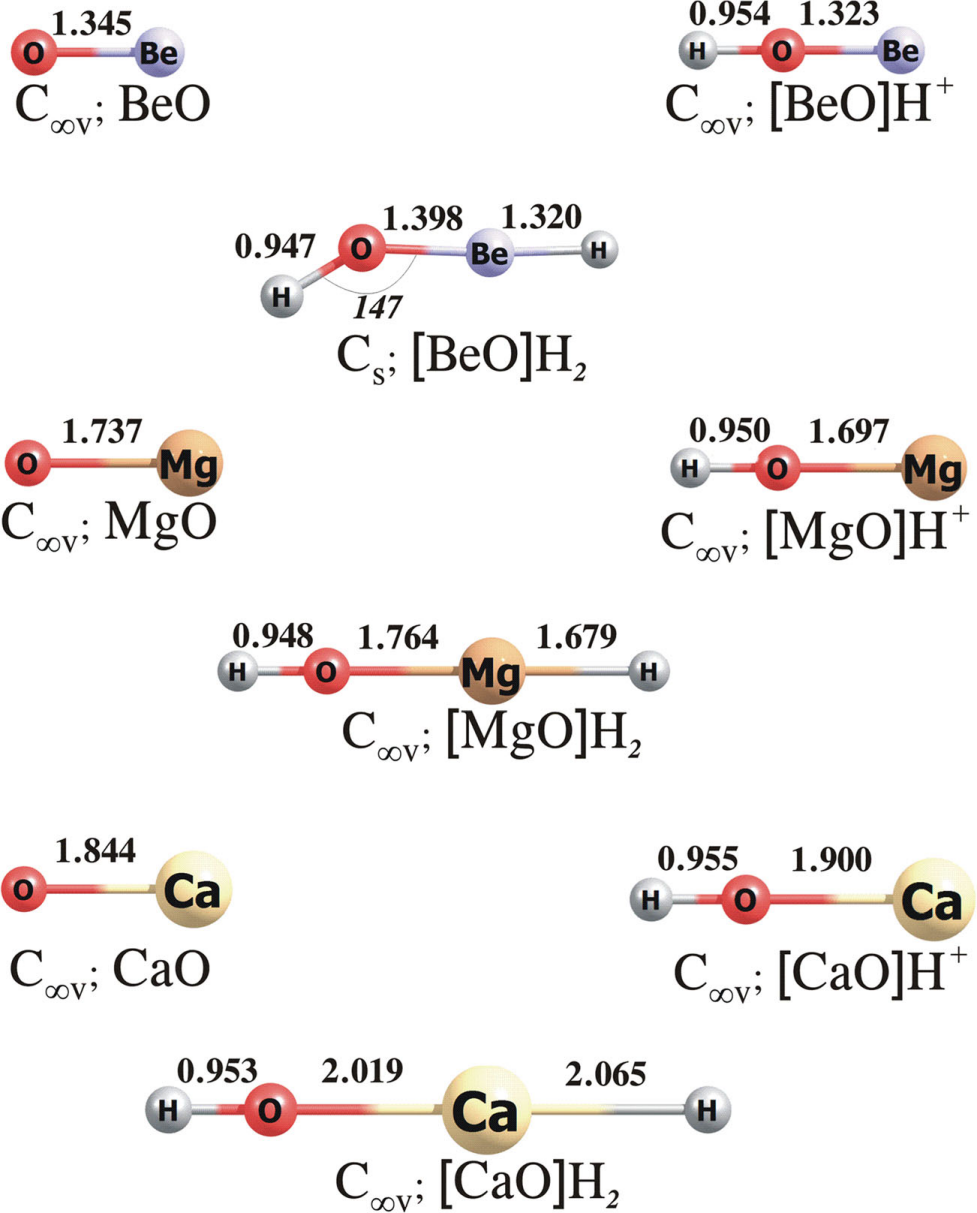
The equilibrium structures of the **M**O, [**M**O]H^+^ and [**M**O]H_2_ (where **M** = Be, Mg, Ca) obtained at the MP2/aug-cc-pVTZ level

**Fig. 2:**
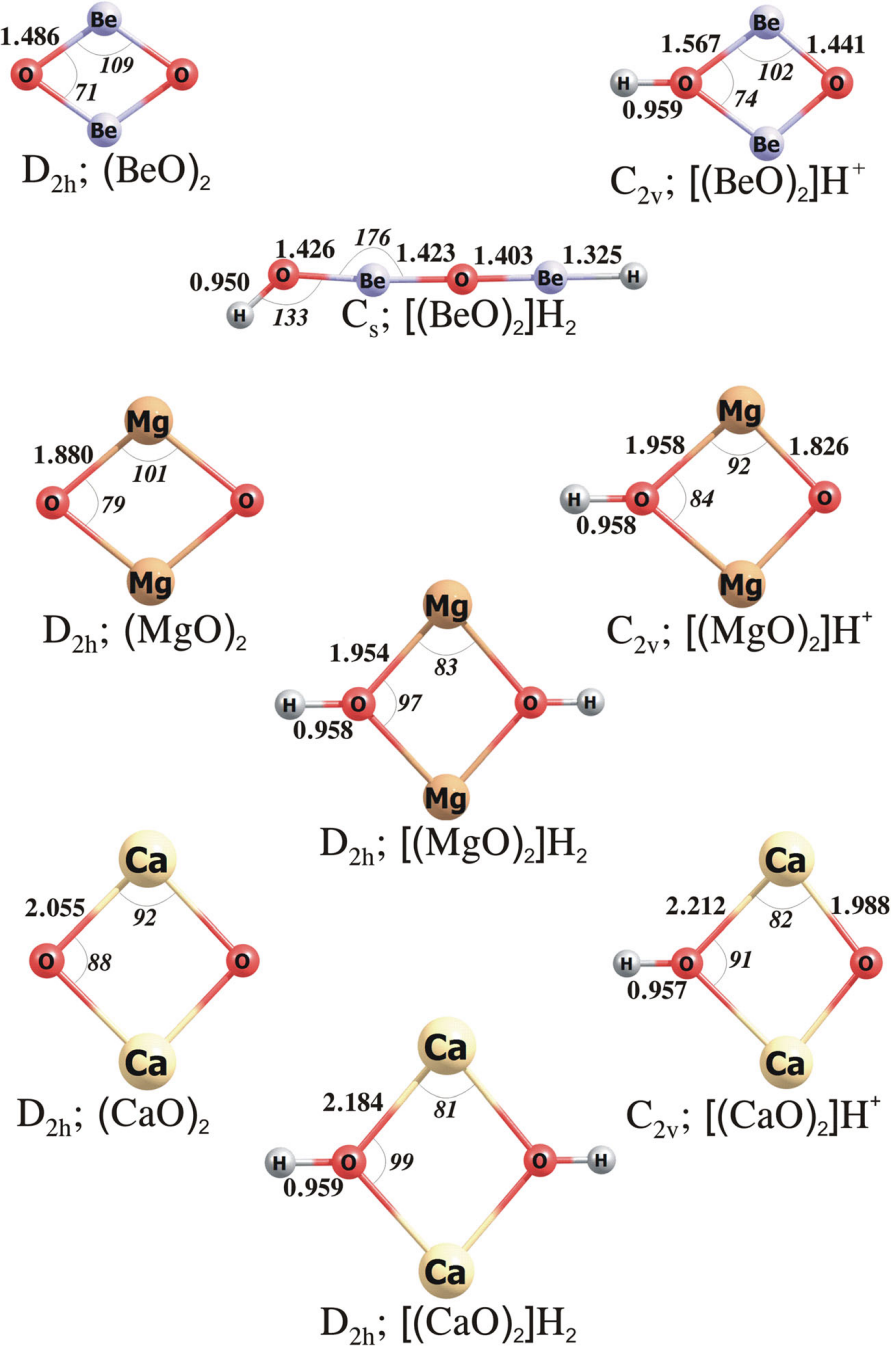
The equilibrium structures of the (**M**O)_2_, [(**M**O)_2_]H^+^ and [(**M**O)_2_]H_2_ (where **M** = Be, Mg, Ca) obtained at the MP2/aug-cc-pVTZ level

The lowest energy structures of **M**O, [**M**O]H^+^, [**M**O]H_2_, (**M**O)_2_, [(**M**O)_2_]H^+^ and [(**M**O)_2_]H_2_ species (**M** = Be, Mg, Ca) are shown in Figs. 1 and 2, whereas the electronic proton affinity and gas-phase basicity of monomeric (**M**O) and dimeric ((**M**O)_2_) alkaline earth metal oxides as well as hydride affinity and gas-phase electrophilicity of the protonated forms of those oxides are gathered in Table 1. According to our findings, the lowest energy isomers of the [**M**O]H^+^ cations correspond to the linear C_∞v_-symmetry structures with the hydrogen atom bonded to the oxygen atom, while the analysis of the most stable structures obtained for [**M**O]H_2_ indicates that hydride anion attaches in all cases to the opposite side of [**M**O]H^+^ molecule (i.e. to the **M** atom, see Fig. 1). The H^−^ attachment to [MgO]H^+^ or [CaO]H^+^ affects the structures only slightly as the resulting neutral [MgO]H_2_ and [CaO]H_2_ molecules are linear (C_∞v_-symmetry). In contrast, the addition of H^−^ to the [BeO]H^+^ cation leads to the bent C_s_-symmetry structure of [BeO]H_2_ (with the Be–O–H valence angle of 146.62°).

**Table 1:** The electronic proton affinities (PA in kcal/mol), gas-phase basicities (GPB in kcal/mol) of the **M**O, (**M**O)_2_ and **M**O***N***_2_O as well as the electronic hydride affinity (HA in kcal/mol) and gas-phase electrophilicity (GPE in kcal/mol) of the corresponding protonated forms (i.e. **M**OH^+^, [(**M**O)_2_]H^+^ and [**M**O***N***_2_O]H^+^; where **M** = Be, Mg, Ca; ***N*** = Li, Na, K ). The results are obtained at the CCSD(T)/aug-cc-pVTZ//MP2/aug-cc-pVTZ level

Species	PA	GPB	Species	HA	GPE
BeO	236.3	220.5	[BeO]H^+^	265.8	265.5
MgO	266.8	242.8	[MgO]H^+^	226.8	218.1
CaO	302.5	278.2	[CaO]H^+^	170.3	161.1
(BeO)_2_	220.4	207.9	[(BeO)_2_]H^+^	253.6	245.3
(MgO)_2_	272.4	259.6	[(MgO)_2_]H^+^	200.5	193.3
(CaO)_2_	300.8	288.2	[(CaO)_2_]H^+^	153.8	143.1
BeOLi_2_O	272.0	259.9	[BeOLi_2_O]H^+^	171.8	160.3
BeONa_2_O	297.1	287.5	[BeONa_2_O]H^+^	154.5	140.5
BeOK_2_O	310.1	298.1	[BeOK_2_O]H^+^	142.3	131.4
MgOLi_2_O	290.7	277.6	[MgOLi_2_O]H^+^	166.7	156.9
MgONa_2_O	309.4	297.7	[MgONa_2_O]H^+^	154.8	144.8
MgOK_2_O	321.7	308.9	[MgOK_2_O ]H^+^	151.7	138.7
CaOLi_2_O	320.2	308.9	[CaOLi_2_O]H^+^	147.6	138.3
CaONa_2_O	331.9	320.4	[CaONa_2_O]H^+^	144.1	131.6
CaOK_2_O	333.1	322.2	[CaOK_2_O]H^+^	134.9	122.0

The calculated PAs and GPBs for alkaline earth metal oxides show a systematic growth with an increase of atomic number of **M** (i.e. the largest PA and GPB values correspond to CaO), see Table 1. The experimental PAs and GPBs are available only for MgO (PA = 236.14 kcal/mol; GPB = 229.30 kcal/mol) and CaO (PA = 284.56 kcal/mol; GPB = 277.80 kcal/mol)[27]. The comparison of those values with our calculated PAs and GPBs may indicate that the CCSD(T)/aug-cc-pVTZ theoretical treatment somewhat overestimates the proton affinity and gas-phase basicity of MgO and CaO by 17.9–30.7 and 0.4–13.5 kcal/mol, respectively. However, it is worth to mention that the experimental data available for magnesium and calcium oxides are based on a single report only (describing the measurements performed in 1962) and might be unreliable as such. Hence, we believe that our PA and GPB values are likely more accurate and represent the best estimates of these quantities available.

As far as the hydride affinities and gas-phase electrophilicities of [**M**O]H^+^ are concerned, our calculations indicate that the HA and GPE decrease in the [BeO]H^+^/[MgO]H^+^/[CaO]H^+^ series (i.e. the lowest HA and GPE correspond to [CaO]H^+^). Clearly, the basicity of **M**O is associated with the electrophilicity of [**M**O]H^+^, as the values gathered in Table 1 affirm (i.e. the larger the basicity of **M**O (CaO > MgO > BeO), the smaller the electrophilicity (with respect to H^−^) of the corresponding [**M**O]H^+^ ([CaO]H^+^ < [MgO]H^+^ < [BeO]H^+^)).

The most stable isomers of (**M**O)_2_ correspond to the rhombic D_2h_-symmetry structures, see Fig. 2. The **M**–O distances in the **M**O dimers are longer than those in the corresponding monomers by 0.141, 0.143 and 0.211 Å for **M** = Be, Mg and Ca, respectively. The lowest energy isomers of [(**M**O)_2_]H^+^ correspond to the kite-shaped C_2v_-symmetry structures with the proton attached to one of the oxygen atoms. As shown in Fig. 2, the **M**–O bonds involving the protonated oxygen atom are slightly longer (by ca. 0.08–0.16 Å) than those in the neutral (**M**O)_2_ systems, whereas the remaining **M**–O separations in [(**M**O)_2_]H^+^ are somewhat smaller (by ca. 0.05–0.07 Å) than the **M**–O distances in the corresponding dimeric oxides.

The attachment of H^–^ to [(**M**O)_2_]H^+^ systems may lead to the formation of either C_s_-symmetry chain-like H–O–**M**–O–**M**–H isomer or D_2h_-symmetry rhombic isomer of [(**M**O)_2_]H_2_. As we verified, the most stable isomer of [(BeO)_2_]H_2_ corresponds to the chain structure with the Be–O–H valence angle of 133.13° (while the rhombic structure is higher in energy by 67.7 kcal/mol), and the lowest energy isomers of [(MgO)_2_]H_2_ and [(CaO)_2_]H_2_ correspond to the rhombic structures (whereas the energies of the corresponding chain structures are larger by 4.3 and 16.6 kcal/mol, respectively), see Fig. 2.

Similar to our predictions formulated for the **M**O monomers, our calculations performed for the (**M**O)_2_ systems indicate that the larger the basicity of (**M**O)_2_ the smaller the electrophilicity (with respect to H^−^) of its corresponding protonated [(**M**O)_2_]H^+^ form. In particular, the calculated PA and GPB values for (**M**O)_2_ increase (from 220 to 301 and from 208 to 288 kcal/mol, respectively) with an increase of the atomic number of **M**, whereas the calculated HAs and GPEs of [(**M**O)_2_]H^+^ decrease (from 254 to 154 and from 245 to 143 kcal/mol, respectively) with the **M** atomic number, see Table 1. In order to verify whether the dimerization affects the basicity and electrophilicity of **M**O systems, we compared the PA, GPB, HA and GPE values of (**M**O)_2_ to those of their corresponding **M**O species. We found that (i) the dimerization of BeO decreases the basicity as well as the electrophilicity of its protonated form (as the PA and GPB of (BeO)_2_ are smaller by 12.6–15.9 kcal/mol and the HA and GPE of [(BeO)_2_]H^+^ are smaller by 12.2–20.2 kcal/mol than the corresponding values calculated for BeO and [BeO]H^+^), (ii) the dimerization of MgO increases the basicity as it leads to larger (by 5.6–16.8 kcal/mol) values of PA and GPB, while the HA and GPE values of the corresponding [(MgO)_2_]H^+^ are lower (by ca. 25 kcal/mol) than those predicted for [MgO]H^+^, (iii) the dimerization of CaO decreases the PA only slightly (by 2 kcal/mol) and increases the GPB by 10 kcal/mol, whereas the HA and GPE values decrease (by 17–18 kcal/mol) with respect to those calculated for [CaO]H^+^ (see Table 1). Finally, it should also be mentioned that MgO, (MgO)_2_, CaO and (CaO)_2_ can be classified as superbases, which means that their PAs (in the range of 267–302 kcal/mol) and GPBs (spanning the 243–288 kcal/mol range) are higher than those predicted for the “proton sponge” whose PA and GPB are equal to 245 and 239 kcal/mol, respectively.

Since various metal oxides are used as catalysts in dehydrogenation reactions, we verified the thermodynamic stability of each neutral [**M**O]H_2_ and [(**M**O)_2_]H_2_ system by examining two dissociation channels, namely, the detachment of H_2_ and H_2_O (see Table 2). In fact, determining the susceptibility of these compounds to liberate molecular hydrogen or water molecule provides an insight not only into the thermodynamic stability of the [**M**O]H_2_ and [(**M**O)_2_]H_2_ systems but also on the recovery processes of the studied oxides (when serving as catalysts in dehydrogenation processes). The positive ΔG_r_^298^ values (spanning the 24–87 kcal/mol range and the 38–110 kcal/mol range for the reactions involving [**M**O]H_2_ and [(**M**O)_2_]H_2_, respectively; see Table 2) indicate that the [**M**O]H_2_ and [(**M**O)_2_]H_2_ systems are stable against the detachment of either H_2_ or H_2_O.

**Table 2:** The Gibbs free energies (ΔG_r_^298^ in kcal/mol) of the fragmentation reactions (at *T* = 298.15 K) considered in this work. The results are obtained at the CCSD(T)/aug-cc-pVTZ//MP2/aug-cc-pVTZ level

Fragmentation path	ΔG_r_^298^
[BeO]H_2_ → BeO + H_2_	87.4
[MgO]H_2_ → MgO + H_2_	79.3
[CaO]H_2_ → CaO + H_2_	58.8
[(BeO)_2_]H_2_ → [BeO]_2_ + H_2_	59.6
[(MgO)_2_]H_2_ → [MgO]_2_ + H_2_	59.4
[(CaO)_2_]H_2_ → [CaO]_2_ + H_2_	37.8
[BeOLi_2_O]H_2_ → BeOLi_2_O + H_2_	26.6
[BeONa_2_O]H_2_ → BeONa_2_O + H_2_	34.4
[BeOK_2_O]H_2_ → BeOK_2_O + H_2_	35.8
[MgOLi_2_O]H_2_ → MgOLi_2_O + H_2_	40.9
[MgONa_2_O]H_2_ → MgONa_2_O + H_2_	48.9
[MgOK_2_O]H_2_ → MgOK_2_O + H_2_	53.9
[CaOLi_2_O]H_2_→ CaOLi_2_O + H_2_	53.6
[CaONa_2_O]H_2_ → CaONa_2_O + H_2_	58.4
[CaOK_2_O]H_2_ → CaOK_2_O + H_2_	50.6
[BeO]H_2_ → Be + H_2_O	77.8
[MgO]H_2_ → Mg + H_2_O	23.8
[CaO]H_2_ → Ca + H_2_O	28.3
[(BeO)_2_]H_2_ → Be_2_O + H_2_O	110.0
[(MgO)_2_]H_2_ → Mg_2_O + H_2_O	79.1
[(CaO)_2_]H_2_ → Ca_2_O + H_2_O	60.1
[BeOLi_2_O]H_2_ → BeOLi_2_ + H_2_O	84.5
[BeONa_2_O]H_2_ → BeONa_2_ + H_2_O	73.8
[BeOK_2_O]H_2_ → BeOK_2_ + H_2_O	81.1
[MgOLi_2_O]H_2_ → MgOLi_2_ + H_2_O	55.2
[MgONa_2_O]H_2_ → MgONa_2_ + H_2_O	49.6
[MgOK_2_O]H_2_ → MgOK_2_ + H_2_O	57.1
[CaOLi_2_O]H_2_ → CaOLi_2_ + H_2_O	60.6
[CaONa_2_O]H_2_ → CaONa_2_ + H_2_O	52.3
[CaOK_2_O]H_2_ → CaOK_2_ + H_2_O	52.2

### The **M**O***N***_2_O oxides and their protonated and hydrogenated forms (**M** = Be, Mg, Ca; ***N*** = Li, Na, K)

**Fig. 3:**
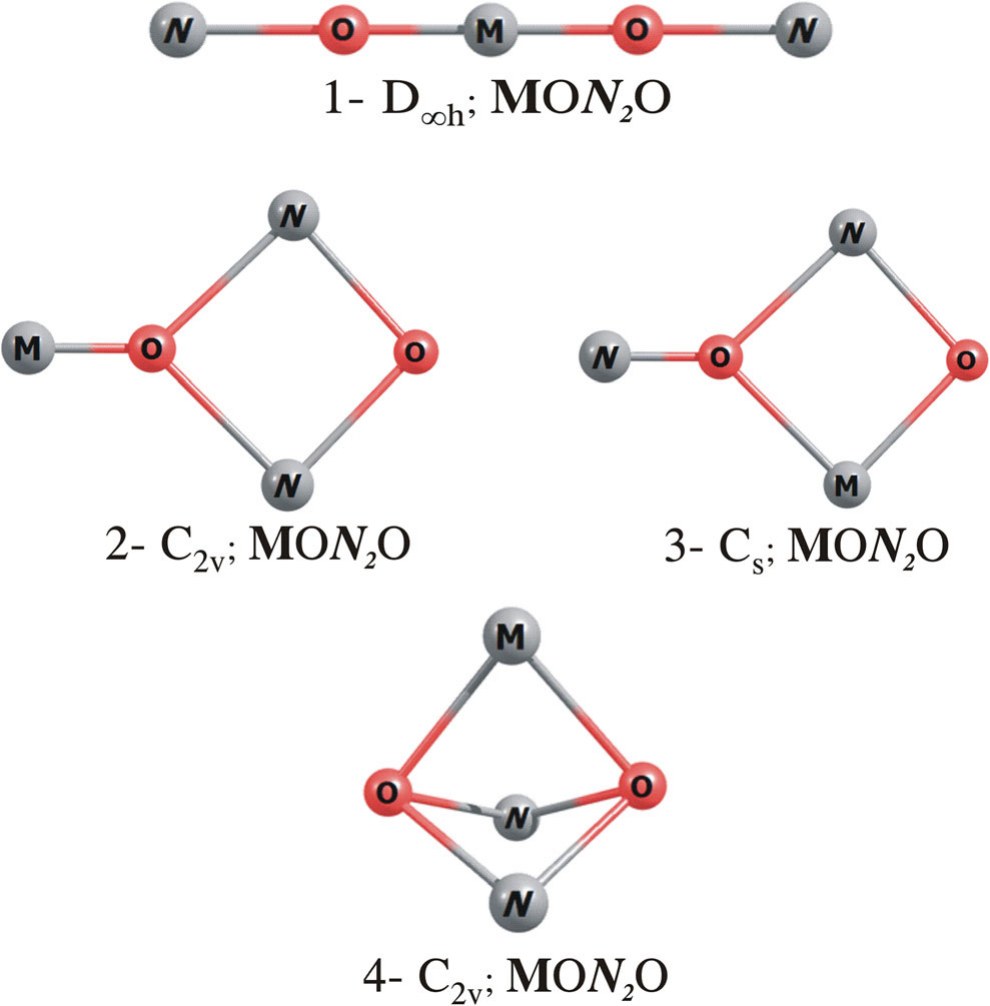
The schematic structures of **M**O***N***_2_O mixed oxides isomers (where **M** = Be, Mg, Ca; ***N*** = Li, Na, K)

An extensive exploration of the ground-state **M**O***N***_2_O potential energy surface of the neutral BeOLi_2_O, BeONa_2_O, BeOK_2_O, MgOLi_2_O, MgONa_2_O, MgOK_2_O, CaOLi_2_O, CaONa_2_O and CaOK_2_O systems matching the **M**O***N***_2_O formula (where **M** and ***N*** stand for alkaline earth metal and alkali metal, respectively) led to four constitutional isomers for each compound considered, see Fig. 3. For each molecule, we verified the thermodynamic stability of its lowest energy isomer by confirming that the ΔG_r_^298^ values calculated for the most likely fragmentation path (i.e. **M**O***N***_2_O→**M**O+***N***_2_O, **M** = Be, Mg, Ca; ***N*** = Li, Na, K) are positive (92–163 kcal/mol). Therefore, we conclude that the mixed **M**O***N***_2_O oxides, once formed, should be stable in gas phase and not susceptible to decomposition yielding **M**O and ***N***_2_O. While exploring the configuration space of the [**M**O***N***_2_O]H^+^ and [**M**O***N***_2_O]H_2_, we performed a random structure search using the previously obtained stationary-point geometries of the **M**O***N***_2_O oxides with a proton or two hydrogen atoms attached as the initial structures. Namely, in order to determine the lowest energy isomeric structures of [**M**O***N***_2_O]H^+^ and [**M**O***N***_2_O]H_2_, various possibilities of attaching the proton or two hydrogen atoms to [**M**O***N***_2_O] were examined by treating them as the starting structures during the independent geometry optimization procedure. Since our main goal was to predict the basicity of **M**O***N***_2_O systems and electrophilicity of their protonated forms as well as to compare those features to the corresponding values predicted for the unmodified **M**O systems, only the lowest energy isomers of BeO***N***_*2*_O, MgO***N***_*2*_O and CaO***N***_*2*_O and their protonated and hydrogenated forms are presented and discussed in the following sections.

#### BeO***N***_2_O, [BeO***N***_2_O]H^+^ and [BeO***N***_2_O]H_2_ (***N*** = Li, Na, K)

**Fig. 4:**
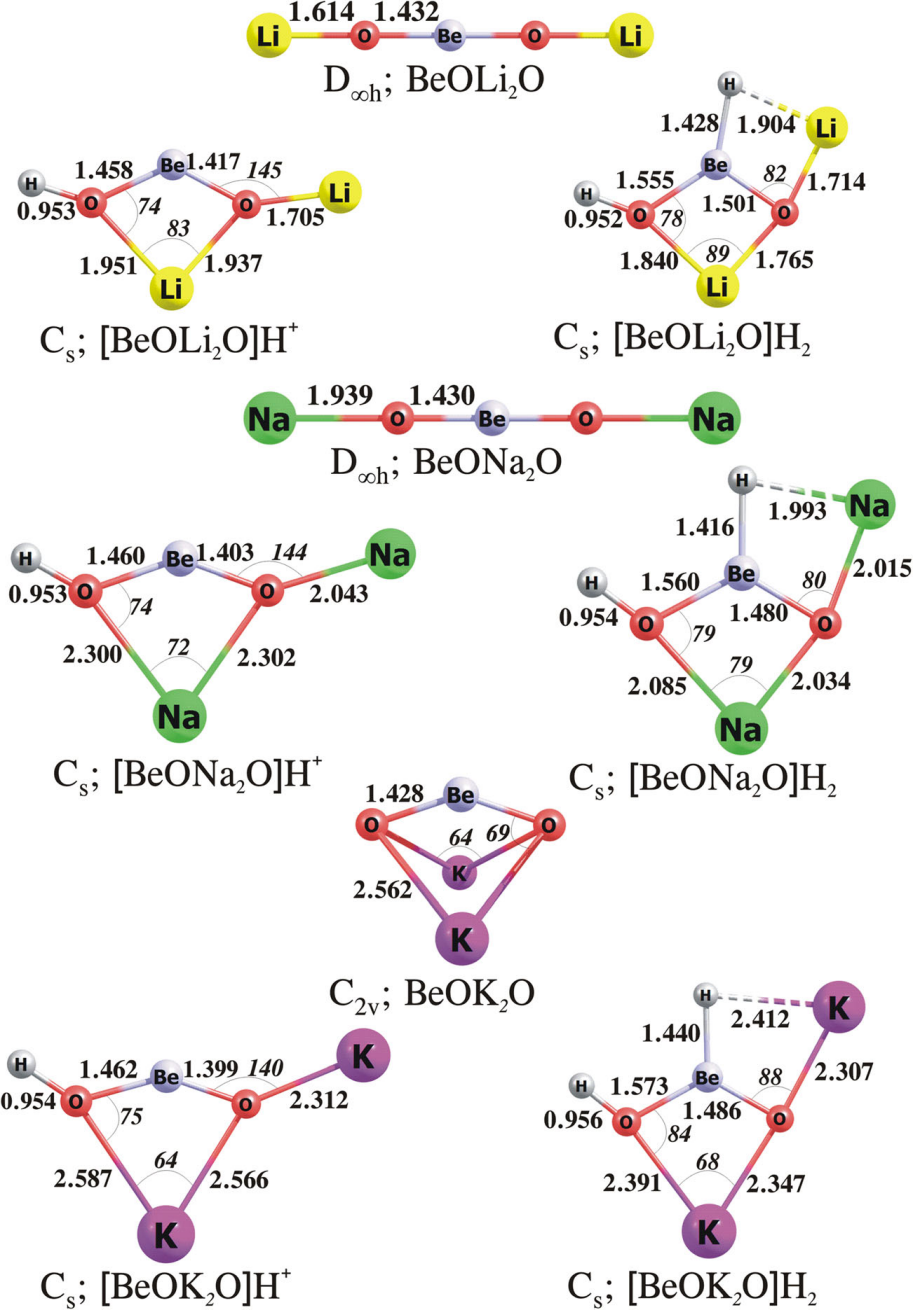
The equilibrium structures of the BeO***N***_2_O, [BeO***N***_2_O]H^+^, [BeO***N***_2_O]H_2_ (where ***N*** = Li, Na, K) obtained at the MP2/aug-cc-pVTZ level

As revealed by our calculations, the most stable isomers of BeOLi_2_O and BeONa_2_O correspond to linear D_∞h_-symmetry structures (labelled **1** in Fig. 3), whereas the lowest energy isomer of BeOK_2_O corresponds to the non-planar compact C_2v_-symmetry structure resembling the system labelled **4** in Fig. 3 (see also Fig. 4 where these global minima are depicted). The second lowest energy isomers (i.e. the compact C_2v_-symmetry BeOLi_2_O and BeONa_2_O and linear D_∞h_-symmetry BeOK_2_O) are higher in energy than the corresponding global minima by ca. 21, 8 and 4 kcal/mol, respectively.

Proton attachment to one of the oxygen atoms of linear BeOLi_2_O and BeONa_2_O structures leads to the bending of the protonated fragment (the resulting Be–O–Li and Be–O–Na valence angles in [BeOLi_2_O]H^+^ and [BeONa_2_O]H^+^ are equal to 129° and 133°, respectively); however, we verified that these bent structures do not correspond to the global minima as their energies are larger by ca. 5 kcal/mol than those predicted for the C_s_-symmetry isomers containing a four-member **M**O_2_***N*** ring with the remaining H and ***N*** atoms bonded to the oxygens (see Fig. 4). As it turned out, the lowest energy isomer of the [BeOK_2_O]H^+^ cation depicted in Fig. 4 corresponds to the similar C_s_-symmetry structure as those of [BeOLi_2_O]H^+^ and [BeONa_2_O]H^+^.

As far as the hydrogenated forms of BeO***N***_2_O are concerned, our calculations indicate that the lowest energy isomers of [BeO***N***_2_O]H_2_ resemble the most stable C_s_-symmetry structures of [BeO***N***_2_O]H^+^ cations with the additional hydrogen atom attached to beryllium atom. The Be–H bond lengths in the [BeO***N***_2_O]H_2_ systems span the 1.416–1.440 Å range and are slightly longer (by ca. 0.1 Å) than those in BeH_2_ (as predicted at the same theory level). As it can be seen from Fig. 4, the addition of H^−^ to the [BeO***N***_2_O]H^+^ systems shortens the ***N***–O bonds (by 0.111–0.268 Å) in the four-membered ring and elongates the Be–O bonds (by 0.077–0.111 Å). Also, the terminal Li, Na and K atoms form the ***N***–H bonds with the nearest H atoms, yet their lengths are larger by 0.316, 0.117 and 0.243 Å with respect to the Li–H, Na–H and K–H bond lengths in the corresponding alkali metal hydrides (as predicted at the same theory level).

As explained above, we view each **M**O***N***_2_O molecule as the alkaline earth metal oxide (**M**O) modified by the alkali metal oxide (***N***_2_O). Hence, the comparison of the PA and GPB of BeO***N***_2_O species as well as the HA and GPE of [BeO***N***_2_O]H^+^ cations to those of the corresponding BeO and [BeO]H^+^ systems allows us to establish the effects caused by this modification. According to our findings, the PA and GPB values increase significantly upon the attachment of the ***N***_2_O to the beryllium oxide. In particular, the PAs and GPBs of the resulting BeOLi_2_O, BeONa_2_O and BeOK_2_O were found to be higher than those of the corresponding BeO by ca. 36–74 and 39–78 kcal/mol, respectively (see Table 1). Moreover, the resulting PAs (272–310 kcal/mol) and GPBs (260–298 kcal/mol) of BeO***N***_2_O systems are larger than the corresponding values of 245 and 239 kcal/mol characterizing the PA and GPB of the “proton sponge”. Hence, we conclude that all BeO***N***_2_O oxides considered might be classified as superbases. On the other hand, a basicity increase of BeO (when modified by the attachment of ***N***_2_O) is associated with the electrophilicity decrease of [BeO]H^+^ (when modified with ***N***_2_O). In particular, the calculated HAs and GPEs of [BeO***N***_2_O]H^+^ span the 142–172 and 131–160 kcal/mol range, respectively, which means that the electrophilicity decreases by ca. 35–49% when the protonated beryllium oxide is mixed with an alkali metal oxide. It is also worth noting that the basicity increase (achieved by attaching ***N***_2_O to BeO) and the electrophilicity decrease (attained by attaching ***N***_2_O to [BeO]H^+^) change in the same direction (i.e. with an increase of the atomic number of ***N***). As a result, we obtained the largest PA/GPB values and the lowest HA/GPE values for BeOK_2_O and its protonated form, respectively (see Table 1).

Finally, we verified the thermodynamic stability of each [BeO***N***_2_O]H_2_ system by examining two possible dissociation channels: dehydration ([BeO***N***_2_O]H_2_→BeO***N***_2_+H_2_O) and dehydrogenation ([BeO***N***_2_O]H_2_→ BeO***N***_2_O+H_2_). Since the ΔG_r_^298^ values determined for these processes turned out to be large and positive (in the 27–84 kcal/mol range), we are confident that all the [BeO***N***_2_O]H_2_ molecules studied are thermodynamically stable. Interestingly, the comparison of the ΔG_r_^298^ values obtained for the fragmentations yielding H_2_O are larger than those predicted for the fragmentations yielding H_2_ which indicates that the latter path should be considered more likely when the decomposition of [BeO***N***_2_O]H_2_ systems is concerned, as the second column in Table 2 affirms.

#### The MgO***N***_2_O, [MgO***N***_2_O]H^+^ and [MgO***N***_2_O]H_2_ systems (***N*** = Li, Na, K)

**Fig. 5:**
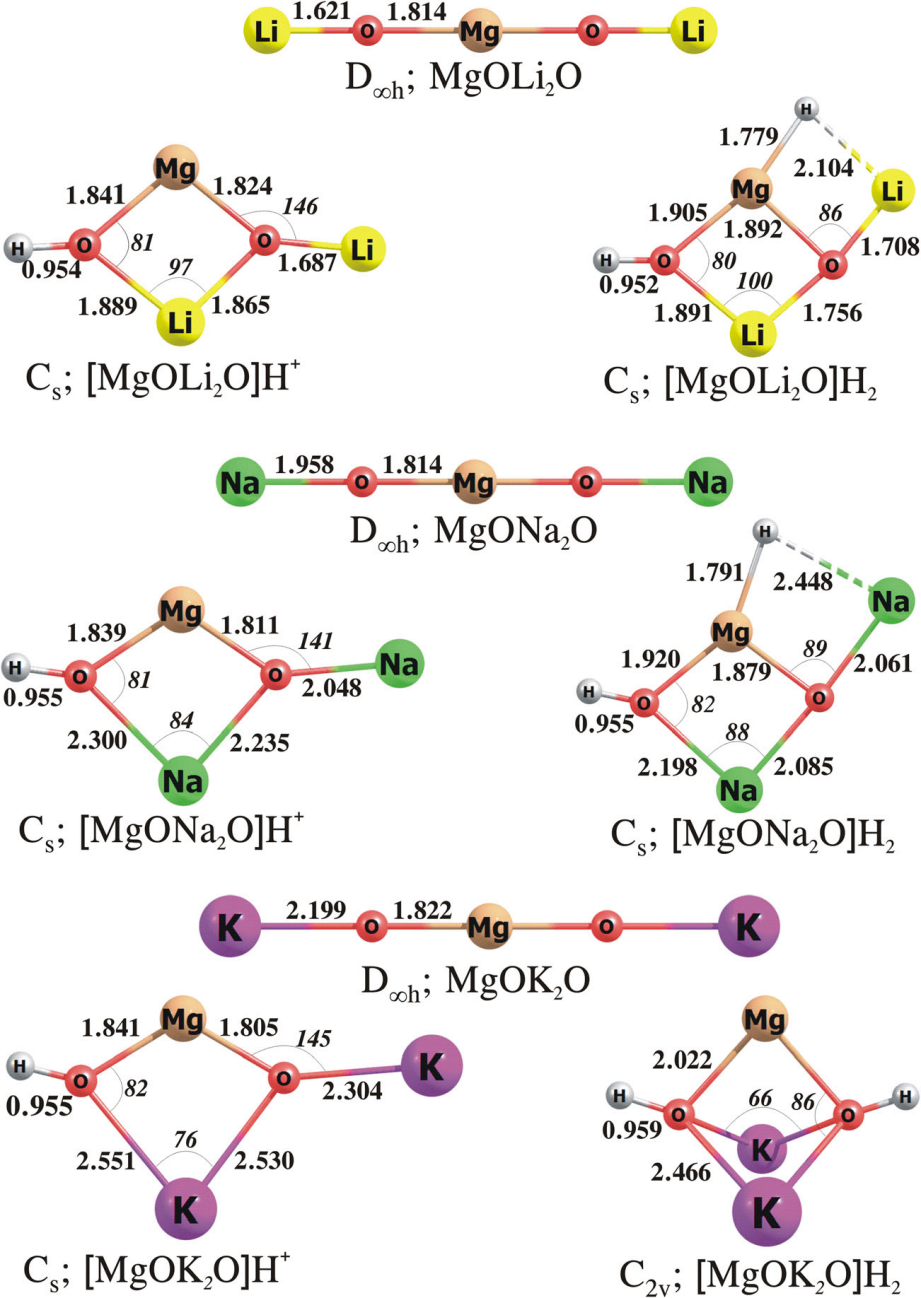
The equilibrium structures of the MgO***N***_2_O, [MgO***N***_2_O]H^+^, [MgO***N***_2_O]H_2_ (where ***N*** = Li, Na, K) obtained at the MP2/aug-cc-pVTZ level

The most stable structures of MgO***N***_2_O, [MgO***N***_2_O]H^+^ and [MgO***N***_2_O]H_2_ involving various alkali metal atoms ***N*** are presented in Fig. 5. As predicted by our calculations, the lowest energy isomers of MgOLi_2_O, MgONa_2_O and MgOK_2_O mixed oxides correspond to the linear D_∞h_-symmetry ***N***O–Mg–O***N*** structures with the magnesium atom localized in the centre bridging two OLi, ONa or OK subunits. The attachment of H^+^ to these compounds leads to the formation of the [MgO***N***_2_O]H^+^ cations whose most stable isomers depicted in Fig. 5 correspond to the structures containing a four-membered MgO_2_***N*** ring with the remaining H and ***N*** atoms bonded to the oxygens. In fact, these global minima of the [MgO***N***_2_O]H^+^ cations closely resemble those found for the [BeO***N***_2_O]H^+^ systems (cf. Figs. 4 and 5). We also confirmed that the relative energies of other isomeric structures of the [MgO***N***_2_O]H^+^ cations (which are not shown in Fig. 5) exceed 8 kcal/mol, thus rendering them uncompetitive near room temperatures.

The structures adopted by the lowest energy isomers of [MgOLi_2_O]H_2_ and [MgONa_2_O]H_2_ resemble those of the corresponding [MgOLi_2_O]H^+^ and [MgONa_2_O]H^+^ cations with the H^−^ attached to the magnesium atom and thus involved in the formation of either Li–H or Na–H bond, see Fig. 5. The Mg–O, O–H, Li–O and Na–O distances in [MgO***N***_2_O]H_2_ (***N*** = Li, Na) differ from those in the [MgO***N***_2_O]H^+^ systems only slightly as the predicted differences do not exceed 0.15 Å. The lengths of the ***N***–H bonds (2.1–2.4 Å) involving terminal ***N*** atoms are substantially larger than those predicted (at the same theory level) for the corresponding isolated alkali metal hydrides (1.588 and 1.876 Å for the Li–H and Na–H, respectively) which indicates relatively weak ***N***–H interactions. In contrast, the lowest energy isomer of [MgOK_2_O]H_2_ adopts the compact C_2v_-symmetry structure with two hydrogen atoms bonded to the oxygens (see Fig. 5). As far as the second lowest energy isomers of [MgO***N***_2_O]H_2_ (***N*** = Li, Na, K) are concerned, we found the C_2v_-symmetry structure of [MgONa_2_O]H_2_ and C_s_-symmetry structure of [MgOK_2_O]H_2_ having their energies only slightly larger (by ca. 2 kcal/mol) than those of their corresponding global minima (thus rendering them competitive near room temperatures) and the C_2v_-symmetry structure of [MgOLi_2_O]H_2_ having its relative energy of ca. 12 kcal/mol.

The data collected in Table 1 indicate that the proton affinity and gas-phase basicity increase when the magnesium oxide is modified by alkali metal oxides. In particular, the PA and GPB values predicted for MgO***N***_2_O systems span the 291–322 and 278–309 kcal/mol range, respectively, whereas the PA of 266.8 kcal/mol and the GPB of 242.8 kcal/mol were calculated for the unmodified MgO. It is also worth to mention that the introduction of alkali metal oxide to magnesium oxide is more effective (with regard to the basicity increase) than the dimerization of MgO, as the PAs and GPBs of MgO***N***_2_O are larger by ca. 18–49 kcal/mol than those of (MgO)_2_. As far as the hydride affinity and gas-phase electrophilicity of [MgO***N***_2_O]H^+^ are concerned, we confirmed that the more basic MgO***N***_2_O the less electrophilic its corresponding protonated form. In the series of MgOLi_2_O/MgONa_2_O/MgOK_2_O, the MgOK_2_O molecule was identified as the most basic (having its PA = 321.7 kcal/mol and GPB = 308.9 kcal/mol), and thus its protonated [MgOK_2_O]H^+^ form exhibits the lowest (among the MgO-based mixed oxides considered) values of HA (151.7 kcal/mol) and GPE (138.7 kcal/mol), see Table 1. The corresponding [MgO***N***_2_O]H_2_ compounds were all found stable with respect to the fragmentations yielding either H_2_ or H_2_O, as indicated by positive Gibbs free energies calculated for those processes (see ΔG_r_^298^ values gathered in Table 2).

#### The CaO***N***_2_O, [CaO***N***_2_O]H^+^ and [CaO***N***_2_O]H_2_ systems (***N*** = Li, Na, K)

**Fig. 6:**
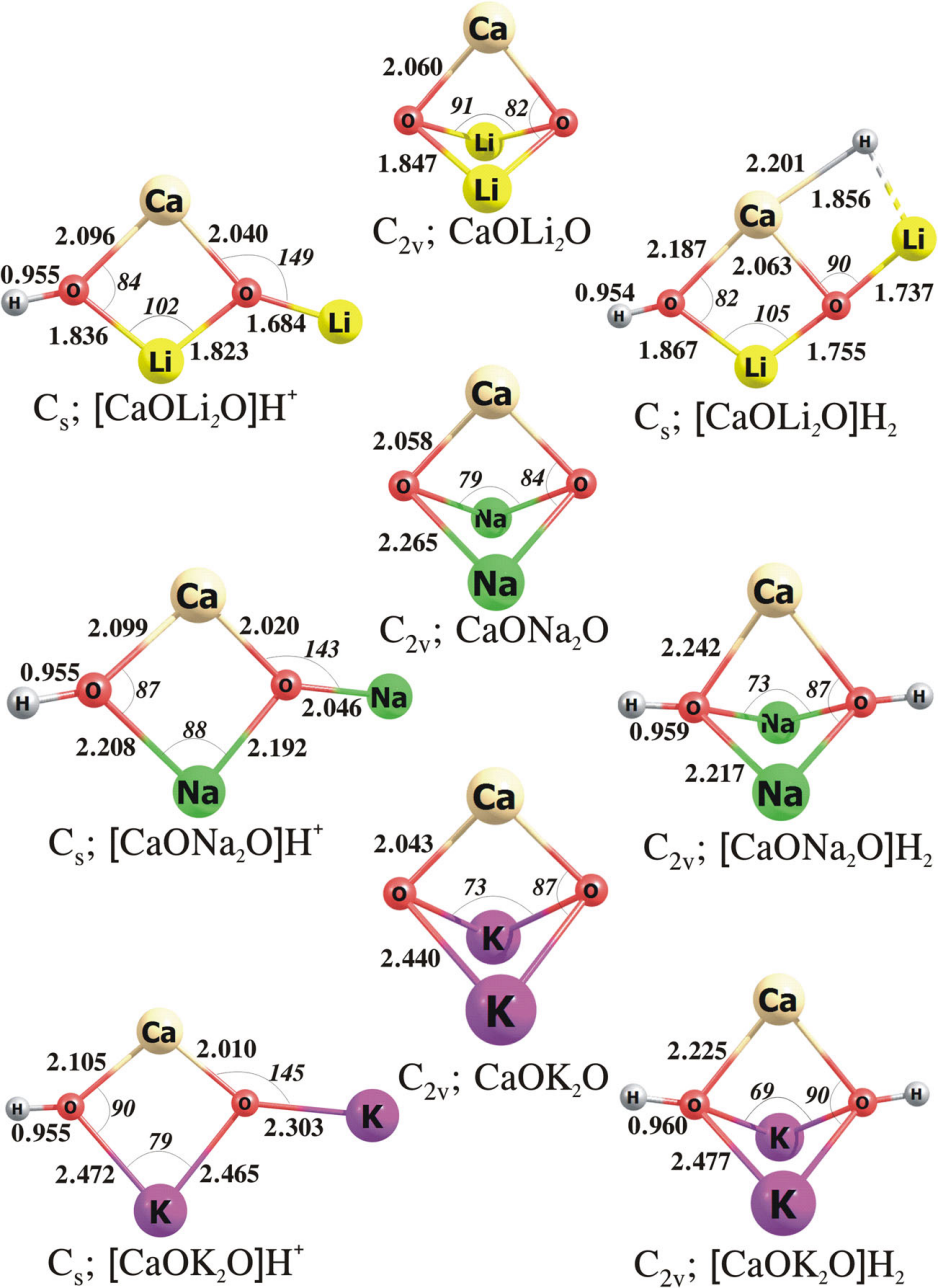
The equilibrium structures of the CaO***N***_2_O, [CaO***N***_2_O]H^+^, [CaO***N***_2_O]H_2_ (where ***N*** = Li, Na, K) obtained at the MP2/aug-cc-pVTZ level

Our calculations revealed that the lowest energy isomers of CaOLi_2_O, CaONa_2_O and CaOK_2_O correspond to the compact C_2v_-symmetry structures, see Fig. 6. We also found that the H^+^ attachment to these structures results in substantial geometry reorganization as the most stable isomers of the [CaO***N***_2_O]H^+^ cations resemble their corresponding [BeO***N***_2_O]H^+^ and [MgO***N***_2_O]H^+^ systems (described in the preceding section, see Figs. 4–6 for comparison). Namely, each [CaO***N***_2_O]H^+^ (***N*** = Li, Na, K) structure is planar (C_s_-symmetry) and contains a tetragonal CaO_2_***N*** frame with the additional H and ***N*** bonded to the oxygen atoms, see Fig. 6.

The lowest energy isomer of [CaOLi_2_O]H_2_ resembles the most stable structure of [CaOLi_2_O]H^+^ cation with the H^−^ anion attached to calcium atom via the elongated (2.201 Å) Ca–H bond (as the Ca–H bond length of 2.048 Å was determined for the CaH_2_ molecule). On the other hand, the most stable isomers of [CaONa_2_O]H_2_ and [CaOK_2_O]H_2_ correspond to the C_2v_-symmetry compact structures with two hydrogen atoms bonded to oxygens (see Fig. 6). We should also mention that the energy of another isomer of [CaONa_2_O]H_2_ (adopting the C_s_-symmetry structure) is larger than the energy of the global minimum by only 0.8 kcal/mol; hence, both these isomers are likely co-existing near room temperatures. In contrast, the relative energy of the second most stable isomer of the [CaOK_2_O]H_2_ compound is considerably larger (8.8 kcal/mol).

The results collected in Table 1 indicate that the introduction of an alkali metal oxide to calcium oxide causes the basicity increase of the latter system. Namely, the PA and GPB values predicted for CaO***N***_2_O (***N*** = Li, Na, K) are higher by ca. 18–31 and 31–44 kcal/mol than those calculated for the isolated CaO molecule. Next, our calculations revealed that as the proton affinity and basicity of CaO***N***_2_O systems increase (with an atomic number of ***N***), the hydride affinity and the electrophilicity of the corresponding [CaO***N***_2_O]H^+^ cations decrease (similar trends were observed for BeO- and MgO-based mixed oxides, see the preceding sections). In particular, the PA and GPB determined for the CaO***N***_2_O mixed oxides increase with an increase of the atomic number of ***N*** (i.e. the highest PA and GPB values correspond to CaOK_2_O), whereas the HA and GPE values predicted for the [CaO***N***_2_O]H^+^ cations decrease in the same direction (i.e. the lowest HA and GPE correspond to [CaOK_2_O]H^+^, see Table 1). It is also worth noting that the [CaOK_2_O]H^+^ is characterized by the lowest HA (134.9 kcal/mol) and the lowest GPE (122.0 kcal/mol) among all [**M**O***N***_2_O]H^+^ cations studied.

Taking into account all the most stable isomers of the [**M**O***N***_2_O]H_2_ (where **M** = Be, Mg, Ca; ***N*** = Li, Na, K) systems, it is worth emphasizing that the appearance of a structure with two hydrogen atoms attached to the oxygen atoms (see [MgOK_2_O]H_2_, [CaONa_2_O]H_2_ and [CaOK_2_O]H_2_ depicted in Figs. 5 and 6) does not exclude the catalytic activity (i.e. serving as a Lewis acid) of the alkali earth metal (**M**). In fact, the appearance of this isomeric structure (as the most stable configuration) is likely related to our approach of determining the Lewis acid strength by evaluating the affinity of a given system to hydride anion. As the H^−^ is characterized by a relatively moderate value of the excess electron binding energy (0.754 eV[40]), its excess negative charge is expected to be effectively delocalized among other more electronegative fragments. Regardless of the structural differences, all calculated HA and GPE values estimated for [**M**O***N***_2_O]H^+^ systems point to a decreasing binding strength of the anionic intermediate (resulting from the bond cleavage, e.g. C–H) with a basicity increase of the corresponding **M**O***N***_2_O mixed oxide.

As far as the thermodynamic stability of the [CaO***N***_2_O]H_2_ systems is concerned, we confirmed that all the hydrogenated CaO-based mixed oxides are stable with respect to fragmentation yielding either H_2_ or H_2_O. In particular, the ΔG_r_^298^ values predicted for the [CaO***N***_2_O]H_2_→CaO***N***_2_O+H_2_ and [CaO***N***_2_O]H_2_→CaO***N***_2_+H_2_O processes are positive (spanning the 51–58 kcal/mol and 52–61 kcal/mol range, respectively) which confirms that these fragmentation channels are practically closed, see Table 2. The CaO***N***_2_O mixed oxide recovery by dehydrogenation is more probable in the case of [CaOLi_2_O]H_2_ and [CaOK_2_O]H_2_ (as the ΔG_r_^298^ values for the H_2_ detachment are less positive than those for the detachment of H_2_O), whereas in the case of [CaONa_2_O]H_2_ the detachment of water molecule turned out to be more achievable (as the ΔG_r_^298^ for H_2_O detachment is less positive (by ca. 6 kcal/mol) than that for the fragmentation yielding H_2_, see Table 2).

## Conclusions

On the basis of our CCSD(T)/aug-cc-pVTZ//MP2/aug-cc-pVTZ calculations performed for (i) alkali earth metal oxides (**M**O) and their protonated and hydrogenated forms ([**M**O]H^+^ and [**M**O]H_2_), (ii) dimers of alkali earth metal oxides ((**M**O)_2_) and their protonated and hydrogenated forms ([(**M**O)_2_]H^+^ and [(**M**O)_2_]H_2_) and (iii) alkali earth metal oxides modified by the attachment of alkali metal oxides (**M**O***N***_2_O) and their protonated and hydrogenated forms ([**M**O***N***_2_O]H^+^ and [**M**O***N***_2_O]H_2_) (where **M** = Be, Mg, Ca; ***N*** = Li, Na, K), we conclude the following:

1. The MgO and CaO oxides can be classified as superbases as their electronic proton affinities (PA) and gas-phase basicities (GPB) are higher than the reference values of 245 and 239 kcal/mol (characterizing the “proton sponge”), whereas the BeO oxide is characterized by smaller PA and GPB values of 236.3 and 220.5 kcal/mol, respectively.
2. The dimerization of MgO and CaO increases (by ca. 10–17 kcal/mol) the basicity of those oxides, whereas the PA and GPB values of BeO decrease (by 16 and 12 kcal/mol, respectively) upon dimerization.
3. The electronic proton affinities (spanning the range of 272–333 kcal/mol) and gas-phase basicities (in the range of 260–322 kcal/mol) predicted for all **M**O***N***_2_O considered are substantially higher than the PA and GPB characterizing the “proton sponge”, which justifies their classification as superbases.
4. The basicity of BeO***N***_2_O, MgO***N***_2_O and CaO***N***_2_O increases with an increase of the atomic number of ***N*** (i.e. the highest PA and GPB values correspond to BeOK_2_O, MgOK_2_O and CaOK_2_O).
5. In the case of magnesium oxide and calcium oxide, the mixing with any alkali metal oxide (Li_2_O, Na_2_O or K_2_O) causes a larger PA and GPB increase than the dimerization process.
6. The CaOK_2_O mixed oxide represents the strongest base described in this contribution (PA = 333.1 kcal/mol, GPB = 322.2 kcal/mol).
7. The electronic hydride affinity (HA) and gas-phase electrophilicity (GPE) values of the [**M**O]H^+^ cations relate to the basicity of their corresponding **M**O molecules (the stronger the **M**O base the less electrophilic its protonated form).
8. The protonated **M**O dimers ([(**M**O)_2_]H^+^) are characterized by even smaller (by ca. 12–26 kcal/mol) values of HA and GPE than their corresponding protonated monomeric forms ([**M**O]H^+^).
9. The electrophilicity of [BeO***N***_2_O]H^+^, [MgO***N***_2_O]H^+^ and [CaO***N***_2_O]H^+^ decreases with an increase of the atomic number of ***N*** (i.e. the lowest HA and GPE values correspond to [BeOK_2_O]H^+^, [MgOK_2_O]H^+^ and [CaOK_2_O]H^+^). The electrophilicity of the [BeO***N***_2_O]H^+^, [MgO***N***_2_O]H^+^ and [CaO***N***_2_O]H^+^ strongly relates to the basicity of the corresponding non-protonated mixed oxides (**M**O***N***_2_O). The **M**OK_2_O represents the strongest base in each **M**OLi_2_O/**M**ONa_2_O/**M**OK_2_O series, and thus its protonated [**M**OK_2_O]H^+^ form is characterized by the lowest values of HA and GPE.
10. The least electrophilic system (with respect to hydride anion) among all species studied is [CaOK_2_O]H^+^ cation (as its HA and GPE values are equal to 134.9 and 122.0 kcal/mol, respectively).
11. All the [**M**O***N***_2_O]H_2_ systems studied are thermodynamically stable species toward the detachment of either H_2_ or H_2_O. However, in most cases (except for [CaONa_2_O]H_2_), the dehydrogenation is less endergonic than the detachment of water molecule which indicates the potential reuse of such species as catalysts in dehydrogenation reactions.

## Data Availability

All data are available on request to the corresponding author.

## References

[1] Gorzawski H, Hoelderich W (1999). Preparation of superbases and their use as catalysts for double-bond isomerization. J Mol Catal A Chem.

[2] Zhang G, Hattori H, Tanabe K (1988). Aldol addition of acetone, catalyzed by solid base catalysts: magnesium oxide, calcium oxide, strontium oxide, barium oxide, lanthanum (III) oxide and zirconium oxide. Appl Catal.

[3] Tsuji H, Yagi F, Hattori H, Kita H (1994). Self-condensation of n-butyraldehyde over solid base catalysts. J Catal.

[4] Gupta J, Papadikis K, Konysheva EY (2021). CaO catalyst for multi-route conversion of oakwood biomass to value-added chemicals and fuel precursors in fast pyrolysis. Appl Catal B Environ.

[5] Shen W, Tompsett GA, Xing R (2012). Vapor phase butanal self-condensation over unsupported and supported alkaline earth metal oxides. J Catal.

[6] Lopez-Olmos C, Morales MV, Guerrero-Ruiz A, Rodríguez-Ramos I (2020) Continuous catalytic condensation of ethanol into 1-butanol: the role of metallic oxides (M = MgO, BaO, ZnO, and MnO) in Cu-M/graphite catalysts. Ind Eng Chem Res. 10.1021/acs.iecr.0c04113

[7] Bancquart S, Vanhove C, Pouilloux Y, Barrault J (2001). Glycerol transesterification with methyl stearate over solid basic catalysts. Appl Catal A Gen.

[8] DOSSIN T, REYNIERS M, MARIN G (2006). Kinetics of heterogeneously MgO-catalyzed transesterification. Appl Catal B Environ.

[9] Ferretti CA, Olcese RN, Apesteguía CR, Di Cosimo JI (2009). Heterogeneously-catalyzed glycerolysis of fatty acid methyl esters: reaction parameter optimization. Ind Eng Chem Res.

[10] Montero JM, Brown DR, Gai PL (2010). In situ studies of structure–reactivity relations in biodiesel synthesis over nanocrystalline MgO. Chem Eng J.

[11] De Sousa FP, Dos Reis GP, Cardoso CC et al (2016) Performance of CaO from different sources as a catalyst precursor in soybean oil transesterification: kinetics and leaching evaluation. J Environ Chem Eng. 10.1016/j.jece.2016.03.009

[12] Corma A, Iborra S, Primo J, Rey F (1994) One-step synthesis of citronitril on hydrotalcite derived base catalysts. Appl Catal A Gen. 10.1016/0926-860X(94)80175-4

[13] Climent MJ, Corma A, Guil-Lopez R et al (1999) Solid catalysts for the production of fine chemicals: the use of ALPON and hydrotalcite base catalysts for the synthesis of arylsulfones. Catal Lett. 10.1023/A:1019075227734

[14] Kabashima H, Tsuji H, Hattori H (1997). Michael addition of methyl crotonate over solid base catalysts. Appl Catal A Gen.

[15] Xu C, Bartley JK, Enache DI et al (2005) High surface area MgO as a highly effective heterogeneous base catalyst for Michael addition and Knoevenagel condensation reactions. Synthesis-Stuttgart. 10.1055/s-2005-918467

[16] Tajbakhsh M, Farhang M, Hosseini AA (2014) MgO nanoparticles as an efficient and reusable catalyst for aza-Michael reaction. J Iran Chem Soc. 10.1007/s13738-013-0338-x

[17] Aramendía MA, Borau V, Jiménez C et al (1996) Magnesium oxides as basic catalysts for organic processes: study of the dehydrogenation-dehydration of 2-propanol. J Catal. 10.1006/jcat.1996.0246

[18] Elkhalifa EA, Friedrich HB (2014). Oxidative dehydrogenation and aromatization of n-octane over VMgO catalysts obtained by using different MgO precursors and different precursor treatments. J Mol Catal A Chem.

[19] Gyngazova MS, Grazia L, Lolli A (2019). Mechanistic insights into the catalytic transfer hydrogenation of furfural with methanol and alkaline earth oxides. J Catal.

[20] Corma A, Iborra S (2006) Optimization of alkaline earth metal oxide and hydroxide catalysts for base-catalyzed reactions. In: Advances in Catalysis. pp 239–302

[21] Tsai TF, Wang FL (2001). Ortho-alkylation of phenol derivatives with methanol over magnesium oxide catalysts. 1. Characterization of promoted magnesium oxide catalysts. Catal Lett.

[22] Védrine JC (2018) Fundamentals of heterogeneous catalysis. In: Metal Oxides in Heterogeneous Catalysis

[23] Jackson SD, Hargreaves JSJ (2008) Metal Oxide Catalysis. Wiley

[24] Gawande MB, Pandey RK, Jayaram RV (2012) Role of mixed metal oxides in catalysis science—versatile applications in organic synthesis. Catal. Sci. Technol

[25] Xue L, He H, Liu C (2009). Promotion effects and mechanism of alkali metals and alkaline earth metals on cobalt−cerium composite oxide catalysts for N 2 O decomposition. Environ Sci Technol.

[26] Nowiak G, Skurski P, Anusiewicz I (2016) Attaching an alkali metal atom to an alkaline earth metal oxide (BeO, MgO, or CaO) yields a triatomic metal oxide with reduced ionization potential and redirected polarity. J Mol Model 22. 10.1007/s00894-016-2955-7 10.1007/s00894-016-2955-7 26994021

[27] Hunter EPL, Lias SG (1998). Evaluated gas phase basicities and proton affinities of molecules: an update. J Phys Chem Ref Data.

[28] Lau YK, Saluja PPS, Kebarle P, Alder RW (1978). Gas-phase basicities of N-methyl substituted 1,8-diaminonaphthalenes and related compounds. J Am Chem Soc.

[29] Møller C, Plesset MS (1934). Note on an approximation treatment for many-electron systems. Phys Rev.

[30] Head-Gordon M, Pople JA, Frisch MJ (1988). MP2 energy evaluation by direct methods. Chem Phys Lett.

[31] Frisch MJ, Head-Gordon M, Pople JA (1990). A direct MP2 gradient method. Chem Phys Lett.

[32] Kendall RA, Dunning TH, Harrison RJ (1992). Electron affinities of the first-row atoms revisited. Systematic basis sets and wave functions. J Chem Phys.

[33] Hill JG, Peterson KA (2017). Gaussian basis sets for use in correlated molecular calculations. XI. Pseudopotential-based and all-electron relativistic basis sets for alkali metal (K–Fr) and alkaline earth (Ca–Ra) elements. J Chem Phys.

[34] Čížek J (2007) On the use of the cluster expansion and the technique of diagrams in calculations of correlation effects in atoms and molecules. In: Advan. Chem. Phys. pp 35–89

[35] Bartlett RJ, Purvis GD (1978). Many-body perturbation theory, coupled-pair many-electron theory, and the importance of quadruple excitations for the correlation problem. Int J Quantum Chem.

[36] Purvis GD, Bartlett RJ (1982). A full coupled-cluster singles and doubles model: the inclusion of disconnected triples. J Chem Phys.

[37] Scuseria GE, Janssen CL, Schaefer HF (1988). An efficient reformulation of the closed-shell coupled cluster single and double excitation (CCSD) equations. J Chem Phys.

[38] Frisch MJ, Trucks GW, Schlegel HE, et al (2016) Gaussian 16. Gaussian, Inc., Wallingford CT

[39] ChemCraft Version 1.6 (build 322)

[40] Lykke KR, Murray KK, Lineberger WC (1991). Threshold photodetachment of H. Phys Rev A.

